# Self-Assembled Polyester Dendrimer/Cellulose Nanofibril Hydrogels with Extraordinary Antibacterial Activity

**DOI:** 10.3390/pharmaceutics12121139

**Published:** 2020-11-25

**Authors:** Yanmiao Fan, Faridah Namata, Johan Erlandsson, Yuning Zhang, Lars Wågberg, Michael Malkoch

**Affiliations:** 1 KTH Royal Institute of Technology, School of Chemical Science and Engineering, Fiber and Polymer Technology, Teknikringen 56-58, SE-100 44 Stockholm, Sweden; yanmiao@kth.se (Y.F.); namata@kth.se (F.N.); jerland@kth.se (J.E.); yunzha@kth.se (Y.Z.); wagberg@kth.se (L.W.); 2 KTH Royal Institute of Technology, Wallenberg Wood Science center. (WWSC), Teknikringen 56-58, SE-100 44 Stockholm, Sweden

**Keywords:** cationic dendrimer, antibacterial materials, carboxylated cellulose nanofibrils, hybrid hydrogels

## Abstract

Cationic dendrimers are intriguing materials that can be used as antibacterial materials; however, they display significant cytotoxicity towards diverse cell lines at high generations or high doses, which limits their applications in biomedical fields. In order to decrease the cytotoxicity, a series of biocompatible hybrid hydrogels based on cationic dendrimers and carboxylated cellulose nanofibrils were easily synthesized by non-covalent self-assembly under physiological conditions without external stimuli. The cationic dendrimers from generation 2 (G2) to generation 4 (G4) based on trimethylolpronane (TMP) and 2,2-bis (methylol)propionic acid (bis-MPA) were synthesized through fluoride promoted esterification chemistry (FPE chemistry). FTIR was used to show the presence of the cationic dendrimers within the hybrid hydrogels, and the distribution of the cationic dendrimers was even verified using elemental analysis of nitrogen content. The hybrid hydrogels formed from G3 and G4 showed 100% killing efficiency towards *Escherichia coli* (*E. coli*), *Staphylococcus aureus* (*S. aureus*) and *Pseudomonas aeruginosa* (*P. aeruginosa*) with bacterial concentrations ranging from 10^5^ CFU/mL to 10^7^ CFU/mL. Remarkably, the hybrid hydrogels also showed good biocompatibility most probably due to the incorporation of the biocompatible CNFs that slowed down the release of the cationic dendrimers from the hybrid hydrogels, hence showing great promise as an antibacterial material for biomedical applications.

## 1. Introduction

Microbial contamination is of great concern in the food processing industry [1], in hospitals [2,3], medical devices [4] and drinking water [5] and causes world-wide losses in the order of billions annually. The emergence of multidrug-resistant (MDR) bacteria makes the situation even more challenging as there are fewer, or even sometimes no, effective antimicrobial agents available for infections caused by these bacteria [6]. Therefore, research focused on developing new antimicrobial materials has gained much attention from both fundamental academic research and industrial applications. Normally, conventional low molecular weight antibiotics have been widely used for treating bacterial infections [7,8]; however, the overuse and misuse of antibiotics have caused the prevalence of antibiotic resistant bacteria [9]. The accumulation of non-degradable antibiotics in the environment also contributes to the evolution of more resistant strains [10]. To solve this issue, one promising way is to develop degradable antimicrobial materials which efficiently kill bacterial while minimizing the risk of triggering the evolution of drug resistant strains [11]. The treatment of surfaces with highly charged cationic polyelectrolytes have shown large promise as non-leaching antibacterial surface treatments [12].

In another context, dendrimers are perfect monodisperse macromolecules with a regular and highly branched three-dimensional (3D) architecture [13,14]. Their unique physicochemical features make them promising candidates in biomedical applications such as drug and gene delivery carriers [15,16], scaffolds for tissue engineering [17,18], biosensors for diagnostics [19] and tissue adhesives [20]. Polyamidoamine (PAMAM) dendrimers, developed by Tomalia and coworkers, have received widespread attention and have been thoroughly investigated in biomedical applications [21,22,23]. Cationic PAMAM dendrimers can enhance the effect of antibiotics and have shown good antibacterial activity against both Gram-negative and Gram-positive bacteria [24,25,26]. However, cationic PAMAM dendrimers display cytotoxicity towards diverse cell lines at high generations and significant mortality in vivo, which limits their applications in biomedical fields [27,28]. Several reports have suggested that the mechanism of amine functional cationic polymers for killing bacteria involves the interaction with the anionic surface of the bacteria and subsequently the disruption of the membrane integrity of the bacteria [29,30,31]. The membrane disrupting mechanism inhibit the development of resistant strains [31]. On the other hand, the polyester dendrimer family based on 2,2-bis(methylol)propionic acid (bis-MPA) is a strong contender in biomedical field due to its facile synthesis, excellent biocompatibility and biodegradability [32,33,34]. Bis-MPA dendrimers have been developed to display cationic β-alanine functionalities with great potential as antibacterial agents, and these dendrimers are completely degradable and exhibit cytotoxicity towards human cells at higher generations [35]. In general, cationic dendrimers often exhibit higher toxicity than anionic and neutral dendrimers [36]. Modification of the cationic dendrimer surfaces with negatively charged or neutral moieties can increase their biocompatibility [37,38]. This is indeed relevant for materials intended for biomedical applications in which more innovative solutions are needed to enhance the biocompatibility of the cationic dendrimers.

Cellulose nanofibrils (CNFs) are bio-based, renewable and non-cytotoxic [39] high aspect ratio nanoparticles which are extracted from wood fibres via combinations of chemical and mechanical treatments [40,41]. By post-treating using the Layer-by-Layer technique [42], CNFs can also be assembled into wet-stable materials such as aerogels [43] and films [44]. For instance, the combination of highly charged nanocellulose and highly charged polyvinylamine has shown high bacterial killing efficiency of *E. coli* and it was shown that a higher surface charge density of the cellulose was beneficial for the bacterial killing efficiency [45]. Furthermore, CNFs also have the ability to form physically crosslinked, low solids content hydrogels of nanoscale dispersed CNFs by adjusting solution parameters such as ionic strength and pH [46,47,48]. Such CNF-based hydrogels have been used for controlled release purposes [49].

Given the ability of CNFs to form stable hydrogels and act as a platform for controlled release of bioactive macromolecules, we herein investigate a series of biocompatible physically crosslinked cationic dendrimer/cellulose nanofibril hybrid hydrogels. The formation of the hybrid hydrogels was induced by the interaction between the cationic dendrimers and anionic charged CNFs. The antibacterial property of the cationic dendrimers and the hybrid hydrogels were explored using both Gram-negative and Gram-positive bacterial strains. The hybrid hydrogels exhibited excellent antibacterial property towards *E. coli*, *S. aureus* and *P. aeruginosa* due to the slow release of cationic dendrimers from the hybrid hydrogels to kill bacteria efficiently. The incorporation of CNFs in the hybrid hydrogels enhanced their biocompatibility. Collectively, the presented results suggested that the hybrid hydrogels are a promising material platform for antibacterial applications.

## 2. Methods

### 2.1. Chemicals

Cesium fluoride and trifluoroacetic acid (TFA) were purchased from Sigma-Aldrich. 2,2-Bis(hydroxymethyl)propionic acid (bis-MPA) and trimethylolpronane (TMP) were obtained from Perstorp AB, Sweden. Boc-β-Alanine was obtained from Alfa Aesar. Carbonyldiimidazole (CDI) was obtained from Carbosynth. Deuterated solvents were purchased from Cambridge Isotope Laboratories. Mueller–Hinton broth (MHB II) was purchased from Fluka. All chemicals and solvents were used as received unless otherwise stated. Never-dried softwood pulp fibres were kindly provided by Domsjö fabriker AB, Örnsköldsvik, Sweden.

*Escherichia coli* 178 (*E. coli* 178) was kindly provided by Professor Paul Orndorff (North Carolina State University). *Staphylococcus aureus* 7920 (*S. aureus* 7920) was kindly provided by Professor Annelie Brauner (Karolinska Institutet), and *Pseudomonas aeruginosa* 22,644 (*P. aeruginosa* 22,644) was obtained from the company DSMZ. Human dermal fibroblast (hDF) and mouse monocyte (Raw 264.7) cells were purchased from the American Tissue Culture Collection (ATCC)

### 2.2. Instruments

Nuclear magnetic resonance (NMR) was performed with the aid of a Bruker Avance instrument (Bruker Biospin, Rheinstetten, Germany). Matrix-assisted laser desorption/ionization-time of flight mass spectrometer (MALDI-TOF) spectra were generated with a Bruker UltraFlex MALDI-TOF with a scout-MTP Ion source (Bruker Daltonics, Bremen, Germany), N_2_-laser (337 nm) and a reflector. Fourier-transform infrared spectroscopy (FT-IR) was performed with a Perkin-Elmer spotlight 400 FTIR system (Waltham, MA, USA) equipped with a single reflection attenuated total reflectance (ATR) in the region of 600–4000 cm^−1^. An ANTEK MultiTek elemental analyzer (PAC, Houston, TX, USA) was used to measure nitrogen content in the hybrid hydrogels. Real-time adsorption of the dendrimers on the CNFs was measured using the Quartz Crystal Microbalance with Dissipation (QCM-D) using an E4 instrument from Q-sense AB (Västra Frölunda, Sweden) with a plasma oven (Harrick PDC-002, Harrick Scientific Corporation, Pleasantville, New York, USA). The total carboxylic acid content of the cellulose fibres was determined by conductometric titration using a Titrino 702 SM titration unit (Metrohm AG, Herisau, Switzerland). Charge density titrations of the fibrils were performed using a ParticleMetrix Stabino^®^ system with Polydiallyldimethylammonium chloride pDADMAC as the titrant for the anionic CNFs and KPVS for the cationic dendrimers.

### 2.3. Dendrimer Synthesis

Cationic amino-functional bis-MPA dendrimers from G2 to G4 (TMP-GX-NH_3_^+^) were synthesized using cesium fluoride promoted esterification chemistry [34,35]. The detailed synthesis can be found in the supporting information.

### 2.4. Preparation of TEMPO Oxidized Fibrils and Preparation of Colloidally Stable Dispersions

TEMPO-oxidized cellulose nanofibrils were prepared using TEMPO-mediated oxidation of dissolving grade pulp at neutral conditions based on a previously published procedure [50], which is an adaptation from the procedure first reported by Saito et al. [51]. A full description of the procedure can be found in the supporting information. The fibrils were subsequently liberated from the fibers by high pressure homogenization using a microfluidizer (M-110EH, Microfluidics Corp, Red Bank, NJ, USA). The colloidally stable CNFs dispersions were then prepared according to previously described procedure [52].

### 2.5. Preparation of the Hybrid Hydrogels

The hybrid hydrogels based on cationic dendrimers and CNFs were prepared similarly to a previously reported procedure with some modifications [47]. Briefly, 66.6 μL of 2 g/L CNFs dispersion (pH 7) was transferred to a cylindrical mold, and 13.4 μL of 50 g/L dendrimer solution (pH 7) was added to the top of the CNFs dispersion aiming for a 1:5 weight ratio of CNFs to dendrimer. The cylindrical mold with diameters of 8 mm was used to prepared hydrogels formed from the 1-mL precursor solution, while the cylindrical mold with a 4 mm diameter was used for hydrogels formed from the 80-µL precursor solution. The gelation time for the 1-mL precursor solution was 24 h while it was 1 h for the 80-µL precursor solution. The formed hybrid hydrogels were washed 3 times with Milli-Q (MQ) water before use. It was hypothesized that the formation of the free-standing hydrogel was due to the diffusion of the dendrimer throughout the CNF dispersion. Nitrogen analysis was used to show the presence of the dendrimer within the hybrid hydrogels.

### 2.6. Characterization of Dendrimers and CNFs

#### 2.6.1. NMR of the Dendrimers

NMR was used to monitor the synthesis and chemical structures of the cationic dendrimers. ^1^H NMR and ^13^CNMR were recorded at 400 MHz and 101 MHz, respectively; ^1^HNMR spectra were acquired using a spectral window of 20 ppm, a relaxation delay of 1 s and 16 scans. ^13^CNMR spectra were acquired using a spectral window of 240 ppm, a relaxation delay of 2 s and 512 scans. Analyses of obtained spectra were conducted using MestreNova version 9.0 (Mestrelab Research S.L 2014).

#### 2.6.2. MALDI-TOF of the Dendrimers

MALDI-TOF was used to monitor the synthesis of the dendrimers as well as to determine the molecular weight of the final products. It was also used to monitor the composition of the dendrimers during the leaching study. MALDI matrices of trans-2-[3-(4-tert-Butylphenyl)-2-methyl-2-propenylidene] (DCTB) and 2,5-Dihydroxybenzoic acid were used for non-polar and polar samples, respectively. The MALDI was calibrated using SpheriCal^®^. The spectra were processed and analyzed with FlexAnalysis version 2.2 from Bruker daltonics.

#### 2.6.3. Total Charge Determination of the Fibers

Prior to the titration measurement, the carboxyl groups on the fibers were protonated in water at pH 2 for 30 min followed by washing with Milli Q water (MQ water) until the conductivity of the rinsing water was below 5 µs/cm. Approximately 0.1 g (dry weight) fibers were subsequently dispersed in 500 mL MQ water containing 0.1 mM HCl and 2 mM NaCl followed by 15 min of bubbling N_2_ through the solution. The titration was performed using NaOH as titrant while continuously monitoring the conductivity of the dispersion and the carboxylic acid content was calculated from the plateau-region of the titration curve based on a previously published procedure [53].

#### 2.6.4. FTIR of the Dendrimers and Hybrid Hydrogels

FTIR was used to analyze the cationic dendrimers and the hybrid hydrogels. All samples were freeze dried before the measurement. Each spectrum was recorded with 4 cm^−1^ resolution between 600 and 4000 cm^−1^ as an average of 16 scans. All spectra were normalized with respect to the baseline at 2200 cm^−1^. The data were processed using the Perkin-Elmer software Spectrum.

#### 2.6.5. Polyelectrolyte Titration of Dendrimers and CNFs

The charge densities of the cationic dendrimers (G2–G4) and the CNFs were determined by polyelectrolyte titration at pH 7. The total charge of the sample was from the titrant consumed at the equivalence points, extracted at 0 mV streaming potential, in the titration curve. From the charge density of the titrant (pDADMAC and poly (vinyl sulfate) potassium salt) and the total amount of tested sample the mass normalized charge density of the dendrimers and the CNFs could be calculated. The titrations were performed at pH 7 to mimic the conditions under which the hydrogels were formed.

#### 2.6.6. Quartz Crystal Microbalance with Dissipation (QCM-D)

The interaction between the cationic dendrimers and CNFs was evaluated using QCM-D by studying the real-time adsorption of dendrimers on a pre-formed surface of CNFs. Prior to the measurements, the SiO_2_ QCM sensors were cleaned with ethanol and water, followed by the treatment in a plasma oven (Harrick PDC-002, Harrick Scientifc Corporation, USA) for 3 min. The cleaned sensors were immediately mounted in the QCM-D flow cells and a stable baseline was recorded in MQ water. Once a stable baseline was established the CNF surface was formed by the sequential adsorption of a Poly vinylamine (PVAm, add-layer) and the CNFs with washing steps after each step. The adsorption of dendrimer onto CNFs was subsequently studied on never dried CNFs, resembling the formation of the hydrogel, in the QCM. The concentrations of the PVAm and the dendrimer solutions were 0.1 g/L and the concentration of the CNFs dispersion was 0.05 g/L.

#### 2.6.7. Nitrogen Analysis of the Hybrid Hydrogels

The nitrogen content within the hybrid hydrogels was measured using an ANTEK MultiTek elemental analyzer (PAC, Houston, TX, USA). The nitrogen of the sample is oxidized to excited NO_2_ which emits a light quantum as the NO_2_ returns to its ground state. By establishing appropriate calibration curves with a known mass of dendrimer, the amount of dendrimer associated with CNFs in the samples could be determined. To clarify that the dendrimers are evenly distributed within the hybrid hydrogels, the hybrid hydrogels were cut into 3 pieces; top, middle and bottom, freeze dried and measured separately.

#### 2.6.8. Leaching Analysis of the Hybrid Hydrogels

A leaching study of the hybrid hydrogels was conducted in phosphate-buffered saline (PBS) solution (pH 7.4) at 37 °C. The hybrid hydrogels (formed with a total volume of 0.5 mL) were prepared and washed in Milli Q water 3 times, then immersed in 2 mL PBS solution, 20 µL aliquots were collected at different time intervals of 1, 2, 4, 8 and 24 h, and the components of the samples were further analyzed by MALDI-TOF-MS.

#### 2.6.9. Rheology of the Hybrid Hydrogels

The storage moduli of the hybrid hydrogels were measured using a TA rheometer (DHR-2) with a plate/plate geometry equipped with a Peltier plate accessory using a stainless-steel upper geometry (∅ = 20 mm). First, 2 g/L CNFs and 50 g/L cationic dendrimer solutions with a 1:5 weight ratio of CNF to dendrimer (total volume of 160 µL) were added successively, and the upper plate was lowered to a final gap of 500 µm. The thin film hybrid hydrogels were equilibrated for 30 min before the time sweeps using strain (ϒ) = 1% and frequency (ω) = 1 Hz.

### 2.7. Antibacterial Study of the Cationic Dendrimers and the Hybrid Hydrogels

#### 2.7.1. Minimum Inhibitory Concentration (MIC) and Minimum Microbicidal Concentration (MMC) Assays

MIC and MMC assays were used to evaluate the antibacterial activities of the cationic dendrimers towards *E. coli*, *S. aureus* and *P. aeruginosa*. Briefly, the samples were diluted with sterilized MQ water in 96-well plates using the double dilution method. Bacterial solutions at log phase were diluted with MHB II broth to reach the concentration of 10^6^ CFU/mL, then an equal volume of bacterial solution was added into the 96-well plates, yielding a final bacterial concentration of 5 × 10^5^ CFU/mL in each well. The plates were incubated at 37 °C for 18 h with shaking of 250 rpm and the optical density was then measured to determine MIC. For MMC, the same procedure was applied, except that the plate counting method was used at the end to determine the microbicidal concentrations.

#### 2.7.2. Antibacterial Assays of the Hybrid Hydrogels

The antibacterial property of the hybrid hydrogels was tested using the disk diffusion test. Cylindrical hydrogels formed from 80 µL precursor solution were prepared using 1 mL syringe. After washing 3 times with sterilized MQ water, the hybrid hydrogels were placed on the agar plate with a bacterial concentration of 10^7^ CFU/mL. All measurements were performed in triplicate.

The antibacterial property of the cylindrical hybrid hydrogels was also tested in bacterial solution. *E. coli*, *S. aureus* and *P. aeruginosa* were cultured in MHB II broth at 37 °C with shaking of 250 rpm overnight. The bacterial solution at log phase was diluted to a series of concentrations ranging from 10^5^ to 10^7^ CFU/mL. The cylindrical hybrid hydrogels were added into 2 mL sterilized centrifuge tubes (Eppendorf) containing 100 µL bacterial solution, and the centrifuge tubes were transferred into the incubator and incubated at 37 °C for 8 h. After the incubation, the plate counting method was used to calculate the bacterial concentrations in the tubes. Centrifuge tubes without the treatment of hydrogels were used as a positive control. All measurements were performed in triplicate.

### 2.8. Cytotoxicity Assay

A monolayer of human dermal fibroblast (hDF) and mouse monocyte cells (Raw 264,7) were used for the cytotoxicity tests. The cell lines were maintained in tissue culture flasks at 37 °C in 5% CO_2_ with Dulbecco’s Modified Eagle Medium (DMEM), supplemented with 10% (*v/v*) Fetal Bovine Serum, 4 mM L-glutamine, 100 IU/mL penicillin and 100 µ/mL streptomycin. The cells were harvested before 100 µL/1 × 10^4^ cells were seeded per well in 96 well plates for the cytotoxicity test. For the cytotoxicity test of cationic dendrimers, the dendrimers were dissolved in media with desired concentrations and introduced to the cells and incubated for 24 h at 37 °C and 5% CO_2_. Subsequently, 10 µL of AlamarBlue agent was applied and incubated for 4 h at 37 °C in 5% CO_2_. Then the plate was shaken for 20 s, and finally fluorescent intensity was measured at ex/em 560/590 nm. For the assessment of hybrid hydrogels, the hybrid hydrogels formed with 80 µL precursor solution were washed three times with sterilized Milli Q water and transferred to 48-well plates containing cells. The 48-well plates were incubated for 24 h (37 °C, CO_2_ (5%)). After the incubation, AlamarBlue (50 µL) was added and incubation continued for 4 h (37 °C, CO2 (5%)), and the fluorescence intensity was measured at ex/em 560/590 nm. All results are shown as mean ± SD.

## 3. Results and Discussion

### 3.1. Synthesis and Characterization of Cationic Dendrimers and Carboxylated CNFs

**Figure 1:**
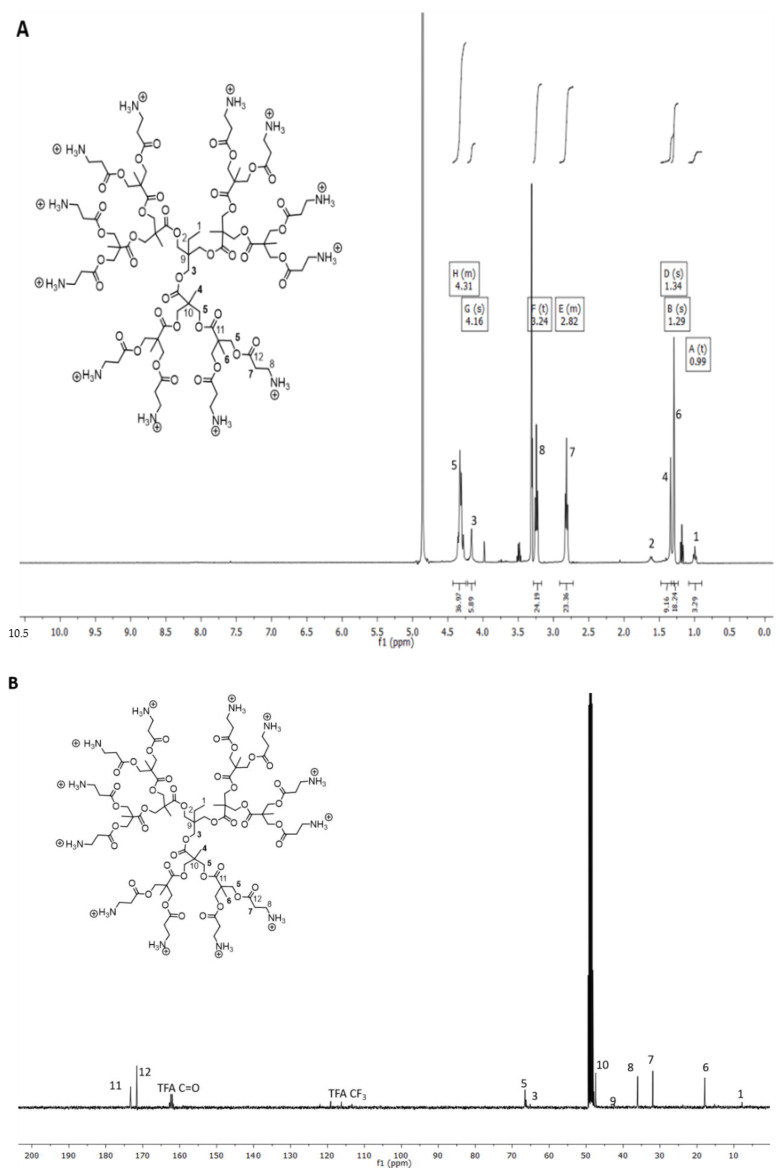
(**A**) ^1^H NMR spectra of generation G2 showing the presence of β-alanine peaks at 3.24 ppm and 2.82 ppm. (**B**) ^13^C NMR spectra of G2 showing the presence of β-alanine peaks at 36.07 ppm and 31.06 ppm in methanol-d_4_.

**Scheme 1:**
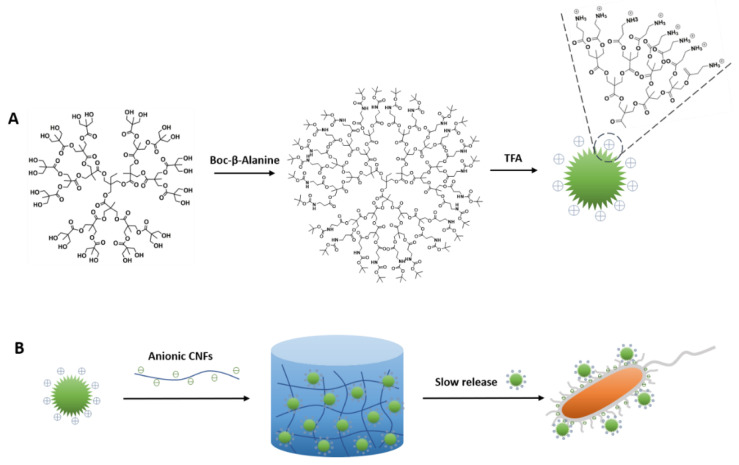
Schematic diagram describing the synthesis of (**A**) boc-β-alanine functional dendrimers (G3) and the deprotection with trifluoroacetic acid (TFA). (**B**) Hybrid hydrogels based on the cationic dendrimers and anionic cellulose nanofibrils (CNFs), and their interaction with bacteria.

The synthesis of cationic dendrimers from G2 to G4 and the subsequent formation of hybrid hydrogels are shown in Scheme 1 and Figure S1. β-alanine terminated dendrimers were synthesized using FPE chemistry [35]. Cationic β-alanine functional dendrimers were achieved through esterification of hydroxy terminated dendrimers with boc-protected β-alanine (Figure S1B) and subsequent deprotection with trifluoroacetic acid (TFA). This resulted in cationic dendrimers as trifluoroacetate salts with high purity and yields of 80% on average. The numbers of peripheral amine groups per molecule of G2, G3 and G4 are 12, 24 and 48, respectively (Figure S1C). NMR was used to confirm the final structure and purity of the dendrimers. Figure 1A shows annotated spectra of ^1^H NMR of G2, and the peaks at 3.24 ppm and 2.82 ppm correspond to β-alanine. ^13^C NMR (Figure 1B) also showed the presence of β-alanine showing peaks at 36.07 ppm and 31.06 ppm, suggesting the functionalization procedure is successful. ^1^H NMR and ^13^C NMR of G3 and G4 are shown in Figures S2 and S3. MALDI-TOF was used to monitor structural defects during the reactions (Figure S4).

Polyelectrolyte titration was used to measure the charge density of both the dendrimers and CNFs adjusted to pH 7.4. The total carboxylic acid content introduced to the pulp fibers through the TEMPO-mediated oxidation was 630 µeq/g before the liberation of the CNFs from the fiber wall by high-pressure homogenization. The surface charge of the liberated fibrils was 550 µeq/g (Table 1). The slightly lower surface charge of the fibrils compared to the total charge of the fibers are presumably due to loss of cellulose chains with a high degree of modification during the dispersion preparation. As for the dendrimers, G2 showed higher charge density compared with G3 and G4 (Table 1), this is probably due to the changing pKa values, which have been extensively studied by other researchers [54,55]. The carboxy to amine ratios are also listed when the weight ratio of CNFs to dendrimers is 1:5. There are 20.7, 15.2 and 15.1 amine groups per carboxy group for G2, G3, and G4, respectively (Table 1).

### 3.2. Hybrid Hydrogel Formation and Characterization

**Figure 2:**
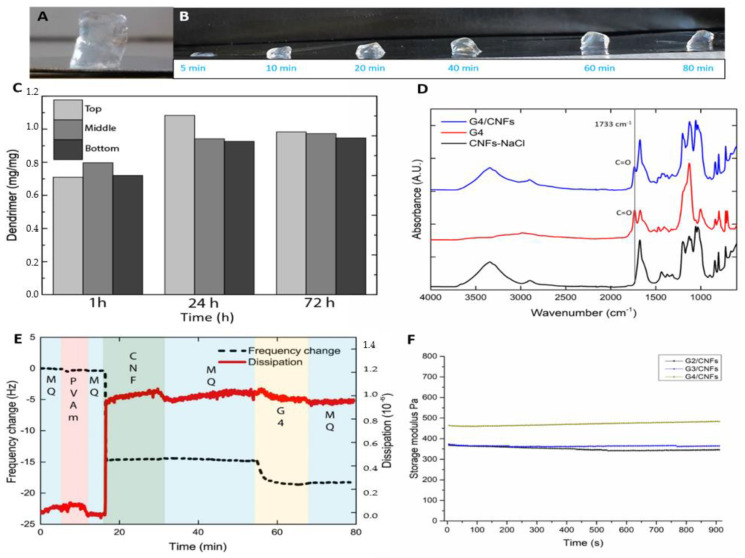
(**A**) Formation of the free-standing G4/CNFs hydrogel. (**B**) Gelation propagation of the hybrid hydrogels with increasing interaction times. (**C**) Display of a homogeneous distribution of cationic dendrimers within the G4/CNFs hydrogel as determined by elemental analysis of the nitrogen content. (**D**) FTIR spectra of G4, G4/CNFs hydrogels and NaCl/CNFs. (**E**) Results from the Quartz Crystal Microbalance with Dissipation (QCM-D) measurements of the interaction of G4 with CNFs. (**F**) The storage moduli of the thin-film hydrogels of G2/CNFs, G3/CNFs and G4/CNFs from the rheology evaluations.

The hybrid hydrogels consisting of anionic CNFs and cationic dendrimer were successfully produced and all hydrogels prepared were macroscopically homogeneous and had sufficient mechanical stability to be free-standing (Figure 2A). In this work, it is hypothesized, similarly to earlier work on CNF-polyampholyte mixes [48], that the observed gelation is due to the adsorption of the cationic dendrimers onto the CNFs which reduces the electrostatic repulsive forces between the CNFs. The reduction in the interparticle repulsion allows the CNFs to form the hydrogels where the CNFs form an interconnected network with the dendrimers in the joints. Since the hydrogels were created via the diffusion of the dendrimers into the CNF dispersion, given that the mixing of cationic polymers and anionic nanoparticles is practically impossible without causing aggregation, the time needed to form the hydrogels as well as the distribution of the dendrimers within the hydrogels needed to be investigated. This is in contrast to the CNF–polyampholyte system [48] where the polyampholyte could be mixed on the nano scale with the CNF dispersion without causing aggregation by simply adjusting the pH. For the CNF–dendrimer hydrogels formed with 80 µL precursor solution, full gelation of the hydrogels was visually observed after 40 min, while there was non-gelled CNF dispersion left when the gelation time was less than 20 min (Figure 2B). The homogeneous distribution of the cationic dendrimers within the hydrogels was determined using an elemental analysis of the nitrogen content since the dendrimers are the only nitrogen source of the hydrogels. The hybrid hydrogels were prepared using different diffusion times (1, 24, 72 h), and these hydrogels were subsequently cut into three sections (top, middle, bottom) and the amount of dendrimers in each section was measured. In general, no significant difference was observed between the top, middle and bottom sections for all hydrogels formed with different times, suggesting a homogeneous distribution of dendrimers (Figure 2C) within all hydrogels. For the hydrogel formed after 1 h, 0.7, 0.8 and 0.7 mg of dendrimers (per mg sample (mg/mg)) were found in the top, middle and bottom sections, respectively. There were similar results for hydrogels formed after 24 h and 72 h, indicating that the homogeneity of the dendrimers can be achieved even after gelation for 1 h. The total amount of dendrimers observed in 72-h hydrogel is higher compared with 1-h hydrogel, this is probably because, when the dendrimers diffuse into the hydrogel and adsorb onto the CNFs, the charge density of the dendrimers and CNFs shift to larger numbers, which results the dendrimers and CNFs can associating to a larger extent. Noteworthy is that the hydrogels were formed at pH 7; there is a large excess of cationic charge originating from the dendrimers compared to the amount of anionic charge from the CNFs. The even distribution of dendrimers and the large excess of cationic charge suggest that the majority of the dendrimers are non-interacting or weakly associated to the CNFs.

Furthermore, FTIR analysis was used to determine the presence of dendrimers within the hybrid hydrogels. The hydrogels formed from 1000 µL precursor solution were freeze dried before the measurement. The peak at 1733 cm^−1^ is a C=O stretch due to carbonyl ester in the dendrimer and was observed in both the hybrid hydrogels (Figure 2D and Figure S5A) and the pure dendrimer sample (Figure 2D), while the CNF hydrogel formed with NaCl [56] (used as control) did not show the dendrimer peak at 1733 cm^−1^ (Figure 2D), indicating the presence of dendrimers within the hybrid hydrogels.

In support of the hypothesis of a gelation induced by the adsorption and subsequent association of the dendrimer-coated CNFs, the interaction between the anionic CNFs and cationic dendrimers was studied by QCM-D. All dendrimers showed an irreversible adsorption of dendrimers onto the CNF decorated QCM surface (Figure 2E and Figure S6), this was indicated as a decreased frequency when the dendrimer was added to the surface, and the frequency change was constant after the subsequent washing with MQ water, indicating the irreversible adsorption of the cationic dendrimers onto the anionic CNFs. The pH of the system was adjusted to pH 7 before the adsorption study to ensure that the reduction in frequency was due to the interaction between the CNFs and dendrimers instead of being affected by changes of the pH or ionic strength. From these results, it can be suggested that the hybrid hydrogels are composed of a physically cross-linked network of CNFs, connected by cationic dendrimer bridges. This is probably with excess dendrimers being contained within the hybrid hydrogels.

From the rheological evaluation of the hydrogels, it is shown that the storage moduli are 343 Pa (G2/CNFs), 365 Pa (G3/CNFs) and 477 Pa (G4/CNFs), respectively (Figure 2F). The loss moduli of the hydrogels are shown in Figure S5B. The G4/CNFs showed the highest storage modulus probably due to that the G4-dendrimer has higher concentration of cationic amine groups on the same molecules leading to a stronger interaction with CNFs.

**Figure 3:**
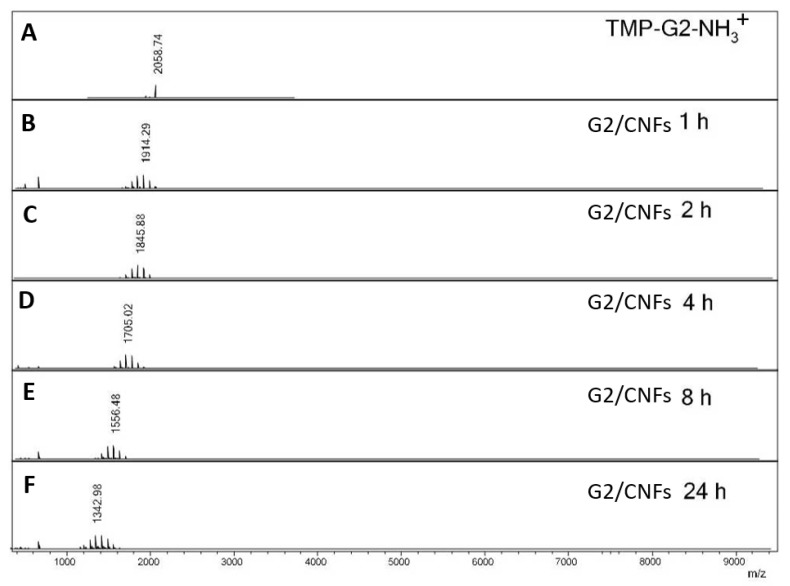
MALDI-TOF spectra of (**A**) pure G2 dendrimer, (**B**) leaching of G2 from the hydrogel after 1 h in phosphate-buffered saline (PBS), (**C**) leaching of G2 from the hydrogel after 2 h in PBS, (**D**) leaching of G2 from the hydrogel after 4 h in PBS, (**E**) leaching of G2 from the hydrogel after 8 h in PBS, (**F**) leaching of G2 from the hydrogel after 24 h in PBS at 37 °C.

A leaching study of dendrimers from the hybrid hydrogels was conducted in PBS solution (pH 7.4) at 37 °C (Figure 3 and Figure S7). Figure 3A shows the MALDI-TOF spectrum of pure G2 dendrimer with a single peak at 2058 m/z. After the incubation of G2/CNFs in PBS for 1 h, multiple peaks appeared in the PBS solution leached out from the hydrogel (Figure 3B), suggesting that some dendrimers and dendrimer fragments were released from the hydrogels. The dendrimers gradually degraded with the increase in time (Figure 3C–E), due to the hydrolysis of the ester bond of the dendrimers in PBS solution.

### 3.3. Antibacterial Testing

#### 3.3.1. MIC and MMC Assays of the Cationic Dendrimers

The antibacterial property of the cationic dendrimers was investigated using MIC and MMC assays. Three types of bacteria were used in the study including *E. coli* (Gram negative), *S. aureus* (Gram positive) and *P. aeruginosa* (Gram negative) since they are very common pathogens. MIC and MMC values were shown in Table 2. All the cationic dendrimers showed good antibacterial property towards all types of bacteria, while TMP-G4-NH_3_^+^ showed the lowest MIC values of 31.25 μg/mL, 62.5 μg/mL, and 31.25 μg/mL towards *E. coli*, *S. aureus* and *P. aeruginosa*, indicating that TMP-G4-NH_3_^+^ is the most efficient cationic dendrimer for killing bacteria. The results also show that higher dendrimer concentrations were needed to kill *S. aureus* than *E. coli* and *P. aeruginosa*, suggesting that those cationic dendrimers are more efficient towards Gram negative strains. The concentrations of amines (NH_3_^+^, μM) needed for inhibiting or killing the bacterial were calculated and the same trend was found for all strains with the following order: TMP-G4-NH_3_^+^ < TMP-G3-NH_3_^+^ < TMP-G2-NH_3_^+^. For example, the active inhibitory or killing concentration of amines towards *E. coli* was 105 μM for TMP-G4-NH_3_^+^, while 214 μM and 441 μM were needed for TMP-G3-NH_3_^+^ and TMP-G2-NH_3_^+^, indicating that TMP-G4-NH_3_^+^ was the most efficient antibacterial agent. Those results are similar with the antibacterial study of amine functionalized cationic PAMAM [25], where higher generations of PAMAM showed lower MIC values and better antibacterial properties. This is probably due to the fact that TMP-G4-NH_3_^+^ has 48 amines per molecule, and the amines are more densely populated at the periphery of the dendrimer, which will result in a stronger interaction with negatively charged bacterial surface. Surface charge density has been demonstrated as a critical element in designing a surface for maximum kill efficiency [30]. The MIC and MMC values of CNFs are above 2000 μg/mL, suggesting that pure CNFs do not, as expected, have any obvious antibacterial property.

#### 3.3.2. Antibacterial Property of the Hybrid Hydrogels

**Figure 4:**
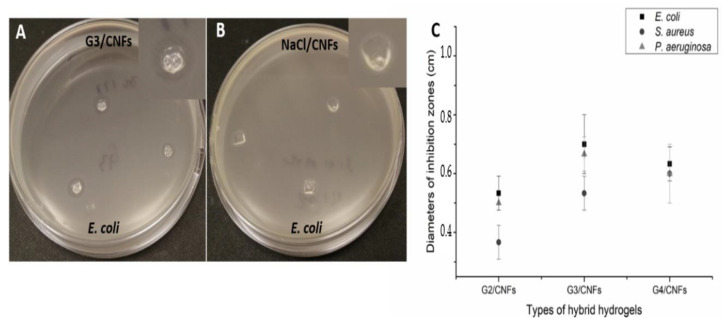
The disk diffusion test of (**A**) G3/CNFs towards *E. coli*, (**B**) NaCl/CNFs towards *E. coli*, (**C**) average diameters of inhibition zones of the hybrid hydrogels against *E. coli*, *S. aureus* and *P. aeruginosa.*

The antibacterial activity of the hybrid hydrogels was tested against *E. coli*, *S. aureus* and *P. aeruginosa* using the disk diffusion test with bacterial concentrations of 10^7^ CFU/mL. Figure 4A and Figure S8 showed the inhibition zones of the three types of hybrid hydrogels after the incubation at 37 °C for 24 h. All hybrid hydrogels showed antibacterial property with visible inhibition zones on the agar plate (Figure 4A and Figure S8), which suggested that active components were released from the hybrid hydrogels to inhibit bacterial growth on the agar plates, while no inhibition zone can be seen from the NaCl/CNFs (Figure 4B). The leaching study indeed showed that the dendrimers were leaching out from the hybrid hydrogels, and gradually degraded into smaller residues. The average diameters of the inhibition zones were measured and presented in Figure 4C. G3/CNFs showed the biggest inhibition zones towards *E. coli* (0.70 cm) and *P. aeruginosa* (0.67 cm), indicating its better antibacterial activity towards those two strains. G4/CNFs showed the average diameter of inhibition zones of 0.60 cm towards *S. aureus*, which is higher than G2/CNFs and G3/CNFs with 0.37 cm and 0.53 cm, respectively. The average diameters of inhibition zones of G2/CNFs are smaller than G3/CNFs and G4/CNFs. Considering that the leaching study of the three types of hydrogels showed a similar degradation rate and leaching rate, TMP-G4-NH_3_^+^, TMP-G3-NH_3_^+^ as well as their residues are more effective at killing bacteria.

**Figure 5:**
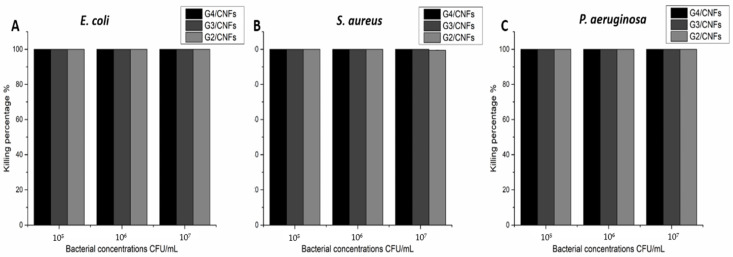
Killing efficiency of the hybrid hydrogels towards different concentrations of (**A**) *E. coli*, (**B**) *S. aureus*, (**C**) *P. aeruginosa* after the incubation for 8 h.

The antibacterial property of the hybrid hydrogels was also tested in 100 µL culture medium containing different bacterial concentrations of 10^5^ CFU/mL, 10^6^ CFU/mL and 10^7^ CFU/mL. After the incubation with the hybrid hydrogels at 37 °C for 8 h, the bacterial solution was diluted, and plate counting method was used to calculate the bacterial concentrations. The results are shown in Figure 5. G4/CNFs and G3/CNFs showed 100% killing ability towards *E. coli*, *S. aureus* and *P. aeruginosa* with all the tested bacterial concentrations, which indicates their excellent antibacterial property. The killing efficiency of G2/CNFs towards *E. coli* and *P. aeruginosa* was 100%, while more than 99.4% of the *S. aureus* was killed. G4/CNFs and G3/CNFs showed better antibacterial property than G2/CNFs towards *S. aureus*, which is consistent with the inhibition zone test where G2/CNFs showed the smallest inhibition zones towards *S. aureus*. Taken together, these results further indicated that the hybrid hydrogels are promising antibacterial materials for treating bacterial infections.

#### 3.3.3. Biocompatibility of the Cationic Dendrimers and Hybrid Hydrogels

**Figure 6:**
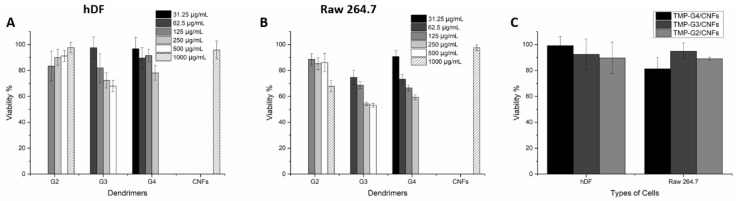
AlamarBlue assay for the cytotoxicity evaluation of (**A**) the cationic dendrimers towards human dermal fibroblast (hDF), (**B**) the cationic dendrimers towards Raw 264.7 and (**C**) the hybrid hydrogels towards both hDF and Raw 264.7 after incubation for 24 h.

Minimal cytotoxicity is crucial for biomedical applications when developing new antibacterial materials. The biocompatibility of the dendrimers, CNFs, and the hybrid hydrogels were tested using human dermal fibroblast (hDF) and mouse monocyte cells (Raw 264.7) in vitro. The concentrations of the dendrimers were chosen based on the MIC values to verify if the dendrimers are toxic towards hDF and Raw 264.7 at the concentrations of MIC values and above. For the cationic dendrimers, TMP-G2-NH_3_^+^ showed excellent biocompatibility towards both hDF (Figure 6A) and Raw 264.7 (Figure 6B) at most tested concentrations with cell viability above 83% (based on ISO 10993-5:2009) [57], except for showing cytotoxicity towards Raw 264.7 (cell viability of 67.9%) when treated with the dendrimer concentration of 1000 µg/mL. TMP-G3- NH_3_^+^ showed obvious concentration-related cytotoxicity, the dendrimer showed good biocompatibility towards hDF at the concentrations ranging from 62.5 to 250 µg/mL (cell viability above 72.3%), however when treated with 500 µg/mL, the cell viability of hDF decreased to 67.9%. The toxic effect is more obvious towards Raw 264.7, the cell viabilities were 54.2% and 53% when treated with 250 µg/mL and 500 µg/mL of TMP-G3- NH_3_^+^, respectively, higher cell viability of 74.7% was reached when using the concentration of 62.5 µg/mL. TMP-G4-NH_3_^+^ was non-toxic towards hDF (concentrations ranging from 31.5 to 250 µg/mL) with highest cell viability of 96.8% reached with the concentration of 31.5 µg/mL, and TMP-G4-NH_3_^+^ also showed excellent biocompatibility towards Raw 264.7 (cell viability of 90.8%) at the same concentration. For the cytotoxicity of CNFs, it showed excellent biocompatibility towards both hDF (cell viability of 95.8%) and Raw 264.7 (cell viability of 97.5%) at the high concentration of 1000 µg/mL. Considering the MIC values of the dendrimers, TMP-G4-NH_3_^+^ showed good antibacterial properties and good biocompatibility at the concentrations of MIC values or even higher concentrations, indicating it is promising antibacterial material to treat infections caused by all bacterial strains tested. For TMP-G3- NH_3_^+^, it is only capable of treating the infection caused by *E. coli* while maintaining good biocompatibility. TMP-G2-NH_3_^+^ showed promising antibacterial effects towards *E. coli* and *P. aeruginosa* while showing good biocompatibility.

For the cytotoxicity of the hybrid hydrogels, all hybrid hydrogels showed good biocompatibility towards both hDF (Figure 6C) and Raw 264.7 (Figure 6C), with cell viability above 81.3%. The highest cell viability of 99.2% was achieved with G4/CNFs towards hDF (Figure 6C). Noteworthily, the hybrid hydrogels formed with TMP-G3-NH_3_^+^ were also biocompatible, although the TMP-G3-NH_3_^+^ in aqueous solution is quite toxic at high concentrations. The introduction of biocompatible CNFs enhanced the biocompatibility of the hybrid hydrogels. The cationic dendrimers are more likely to interact with the anionic charged cell surfaces if they can move freely in the aqueous solution, while the formation of the hybrid hydrogels limited the free movement of the cationic dendrimers and their interaction with the cells, thus reducing the cytotoxicity of the materials. The gradual release of dendrimers and their residues from the hybrid hydrogels can kill bacterial efficiently while being compatible towards cells.

## 4. Conclusions

In summary, β-Alanine functional cationic dendrimers from G2 to G4 were synthesized through the esterification reaction. Most cationic dendrimers showed excellent antibacterial properties towards *E. coli*, *P. aeruginosa* and *S. aureus* and good biocompatibility at the MIC values or even higher concentrations. Hybrid hydrogels based on cationic dendrimers and CNFs were developed and carefully characterized, and it could be established that using the dendrimer/CNFs composite, the biocompatibility of the hybrid hydrogels can be significantly improved while maintaining their excellent antibacterial property. This was achieved by the slow release of dendrimers from the hybrid hydrogels, which efficiently kills bacteria while being compatible towards cells. G3/CNFs and G4/CNFs showed excellent antibacterial properties towards *E. coli*, *P. aeruginosa* and *S. aureus* (concentrations of 10^5^ CFU/mL, 10^6^ CFU/mL and 10^7^ CFU/mL) with killing efficiency of 100%, indicating that they are promising antibacterial materials for the treatment of bacterial infections.

## Figures and Tables

**Table 1:** Polyelectrolyte titration of the anionic CNFs and cationic dendrimers.

Generation	Amount Dendrimer	Charge Density (Dendrimers)	Amount CNFs	Charge Density (CNFs)	Carboxy to Amine Ratio	
Unit	mg	µe/g	mg	µe/g	COO^-^	NH_3_^+^
TMP-G2-NH_3_^+^	8.5	2416.0	1.7	550.0	1.0	20.7
TMP-G3-NH_3_^+^	8.5	1775.0	1.7	550.0	1.0	15.2
TMP-G4-NH_3_^+^	8.5	1765.0	1.7	550.0	1.0	15.1

**Table 2:** Minimum inhibitory concentration (MIC) and minimum microbicidal concentration (MMC) values of the cationic dendrimers and CNFs.

Measurement	Bacterial Strains	*E. coli*			*S. aureus*			*P. aeruginosa*		
	Unit	μg/mL	μM	NH_3_^+^	μg/mL	μM	NH_3_^+^	μg/mL	μM	NH_3_^+^
				(μM)			(μM)			(μM)
MIC value	TMP-G2-NH_3_^+^	125	36.8	441	1000	294.1	3529	500	147.1	1765
	TMP-G3-NH_3_^+^	62.5	8.9	214	500	71.3	1710	125	17.8	428
	TMP-G4-NH_3_^+^	31.25	2.2	105	62.5	4.4	211	31.25	2.2	105
	CNFs	>2000			>2000			>2000		
	Unit	μg/mL	μM	NH_3_^+^	μg/mL	μM	NH_3_^+^	μg/mL	μM	NH_3_^+^
				(μM)			(μM)			(μM)
MMC value	TMP-G2-NH_3_^+^	125	36.8	441	4000	1176	14116	500	147.1	1765
	TMP-G3-NH_3_^+^	62.5	8.9	214	1000	142.6	3420	125	17.8	428
	TMP-G4-NH_3_^+^	31.25	2.2	105	125	8.8	422	31.25	2.2	105
	CNFs	>2000			>2000			>2000		

## Supplementary Materials

The following are available online at https://www.mdpi.com/1999-4923/12/12/1139/s1, Figure S1. (A) Structures of hydroxy functional dendrimers from G2 to G4. (B) Synthetic procedures for the cationic dendrimer (G3). (C) Structures of cationic dendrimers from G2 to G4; Figure S2. (A) 1H and (B) 13C NMR spectra of G3 in methanol-d4; Figure S3. (A) 1H and (B) 13C NMR spectra of G4 in methanol-d4; Figure S4. MALDI-TOF spectra of cationic dendrimers from G2 to G4; Figure S5. (A) FTIR spectra of G2/CNFs, G3/CNFs and G4/CNFs showing dendrimer peak at 1733 cm-1. (B) The loss moduli of the thin-film hydrogels of G2/CNFs, G3/CNFs and G4/CNFs from the rheology evaluations; Figure S6. QCM-D of the interaction of CNFs with (A) G2 and (B) G3; Figure S7. Leaching out study of G3/CNFs and G4/CNFs using MALDI-TOF; Figure S8. The disk diffusion test of the hybrid hydrogels using a bacterial concentration of 107 CFU/mL, and NaCl/CNFs hydrogels as the control.
