# Supplementary material for: Self-Assembled Polyester Dendrimer/Cellulose Nanofibril Hydrogels with Extraordinary Antibacterial Activity
Source: Pharmaceutics. 2020 Nov 25;12(12):1139. doi: 10.3390/pharmaceutics12121139 (PMC7761394; doi:10.3390/pharmaceutics12121139)
Supplement: Supplementary file 1 [file pharmaceutics-12-01139-s001.pdf]

pharmaceutics

Supplemetary Materials: Self-Assembled Polyester Dendrimer/Cellulose Nanofibril Hydrogels with Extraordinary Antibacterial Activity

Yanmiao Fan, Faridah Namata, Johan Erlandsson, Yuning Zhang, Lars Wågberg and Michael Malkoch

TMP-G2-OH, TMP-G3-OH, TMP-G4-OH were synthesised based on a previously published procedure [1].

General procedure for synthesizing cationic functional dendrimers from hydroxy terminated dendrimers

Boc-protected β-alanine (1.5 eq/OH group) was dissolved in ethyl acetate at 1 M concentration at 50 °C. CDI (1.5 eq/OH group) was slowly added to active carboxyl groups and the reaction was monitored with ^{1}H NMR. Upon activation completion, hydroxy functional dendrimer (1 eq) and cesium fluoride (0.2 eq/OH group) were added and the reaction was left overnight. The reaction mixture was quenched with water, diluted with ethyl acetate and washed 4 times with 10% $NaHSO_4$, 4 times with 10% $NaHCO_3$ and once with brine. The organic phase was then dried over $MgSO_4$. The solvent was removed under vacuum, and boc-protected β-alanine functional dendrimers (TMP-Gx-NHBoc) were collected as white powder. TMP-Gx-NHBoc was transferred to a round bottom flask and trifluoroacetic acid was dropwise added until no more bubbles evolve in the flask. Methanol was added to the flask and excess trifluoroacetic acid was removed using the rotatory evaporator, and this step was repeated 4 times. The reaction mixture was precipitated in ether, decanted and the trace solvent was removed under vacuum. β-alanine terminated dendrimers were collected as sticky white solid.

TMP-G2-NH_3^+: Boc-protected β-alanine (5.69 g, 30.07 mmol, 18 eq.), CDI (4.88 g, 30.07 mmol, 18 eq.), TMP-G2-OH (2 g, 1,67 mmol, 1 eq.), cesium fluoride (0.61 g, 4.01 mmol, 2.4 eq.) were reacted giving TMP-G2-NHBoc (4.92 g, 1.52 mmol, 91% yield). TMP-G2-NHBoc (2 g, 0.62 mmol, 1eq.) was deprotected obtaining TMP-G2-NH_3 (1.01 g, 0.50 mmol, 81%) as a sticky white solid. ^{1}H NMR (400 MHz, Methanol-d4) δ 4.43 – 4.24 (m, 36H), 4.16 (s, 6H), 3.24 (t, J = 6.7 Hz, 24H), 2.91 – 2.72 (m, 24H), 1.62 (d, J = 7.6 Hz, 2H), 1.34 (s, 9H), 1.29 (s, 18H), 0.99 (t, J = 7.5 Hz, 3H).^{13}C NMR (101 MHz, MeOD) δ 173.30, 171.60, 162.02, 116.23, 66.56, 66.25, 47.42, 42.47, 36.07, 31.96, 17.90, 17.87, 7.81. FT-IR (cm^{-1}): 3463, 3002 (br., N-H), 1731 (C=O), 1671, 1628, 1520, 1474, 1408, 1325, 1176, 1128, 1057, 1006, 836, 797, 722, 708. MALDI: $[M]_{Calc}$: 2030.97 Da $[M]_{Found}$: 2057.70 Da

TMP-G3- NH_3^+: Boc-protected β-alanine (5.30 g, 27.99 mmol, 36 eq.), CDI (4.54 g, 27.99 mmol, 18 eq.), TMP-G3-OH (2 g, 0.77 mmol, 1 eq.), cesium fluoride (0.57 g, 3.73 mmol, 4.8 eq.) were reacted giving TMP-G3-NHBoc (4.56 g, 0.68 mmol, 88% yield). TMP-G3-NHBoc (2 g, 0.30 mmol, 1eq.) was deprotected obtaining TMP-G3-NH_3 (1.12 g, 0.26 mmol, 87%) as a sticky white solid. ^{1}H NMR (400 MHz, Methanol-d_4) δ 4.32 (dt, J = 18.3, 7.5 Hz, 79H), 4.21 – 4.18 (m, 6H), 3.25 (t, J = 6.7 Hz, 43H), 2.83 (t, J = 6.8 Hz, 43H), 1.63 (d, J = 7.8 Hz, 2H), 1.42 – 1.22 (m, 51H), 0.99 (t, J = 7.5 Hz, 3H).^{13}C NMR (101 MHz, MeOD) δ 173.53, 173.29, 171.87, 162.46, 116.40, 66.72, 66.39, 47.62, 36.31, 32.21, 18.20. FT-IR (cm^{-1}): 3404, 2997 (br., N-H), 1736 (C=O), 1674, 1625, 1517, 1471, 1403, 1325, 1173, 1123, 1056, 1003, 839, 800, 724, 705. MALDI: $[M]_{Calc}$: 4275.98 Da $[M]_{Found}$: 4284.57 Da

TMP-G4- NH_3^+: Boc-protected β-alanine (5.09 g, 26.88 mmol, 72 eq.), CDI (4.36 g, 26.88 mmol, 72 eq.), TMP-G3-OH (2 g, 0.37 mmol, 1 eq.), cesium fluoride (0.54 g, 3.58 mmol, 9.6 eq.) were reacted giving TMP-G4-NHBoc (4.21 g, 0.31 mmol, 83% yield). TMP-G4-NHBoc (2 g, 0.15 mmol, 1eq.) was deprotected obtaining TMP-G4-NH_3 (0.98 g, 0.11 mmol, 76%) as a sticky white solid. ^{1}H NMR (400 MHz, Methanol-d_4) δ 4.31 (q, J = 11.6, 11.1 Hz, 222H), 3.27 (t, J = 6.7 Hz, 93H), 2.95 – 2.79 (m, 96H), 1.45 – 1.22 (m, 158H). ^{13}C NMR (101 MHz, MeOD) δ 173.55, 1z73.32, 171.94, 161.86, 116.27, 66.69, 47.60, 36.33, 32.23, 18.26. FT-IR (cm^{-1}): 3477, 3028 (br., N-H), 1736 (C=O), 1674, 1630, 1520, 1470, 1408,1330, 1130, 1056,1002, 839, 800, 722, 708. MALDI: $[M]_{Calc}$: 8766.01 Da $[M]_{Found}$:8656.55 Da

TEMPO procedure

The fibers were prepared following a previously published literature [2]. Briefly, the never-dried pulp was preliminary washed to remove ions and replace with sodium counterions. It was then further purified to remove residue lignin and impurities. Pulp (26 g) was washed with an acid solution (HCl) and a basic solution ($NaHCO_3$) for 20 minutes each. For the purification step, the pulp was washed in the acetate buffer solution with $NaClO_2$. For the TEMPO oxidation, the pulp was added to a 0.1 M phosphate buffer solution (260 mL) at 60 °C equipped with TEMPO (4.1 g), $NaClO_2$ (29.4 g) and NaClO (13.8 g). The reaction mixture was stirred for 140 minutes, filtered and washed repeatedly with Milli-Q water. The oxidised fibres were diluted and mechanically disintegrated in a high pressure Microfluidizer (M-110EH, Microfluidics Corp, US) resulting in TEMPO-oxidised CNFs.

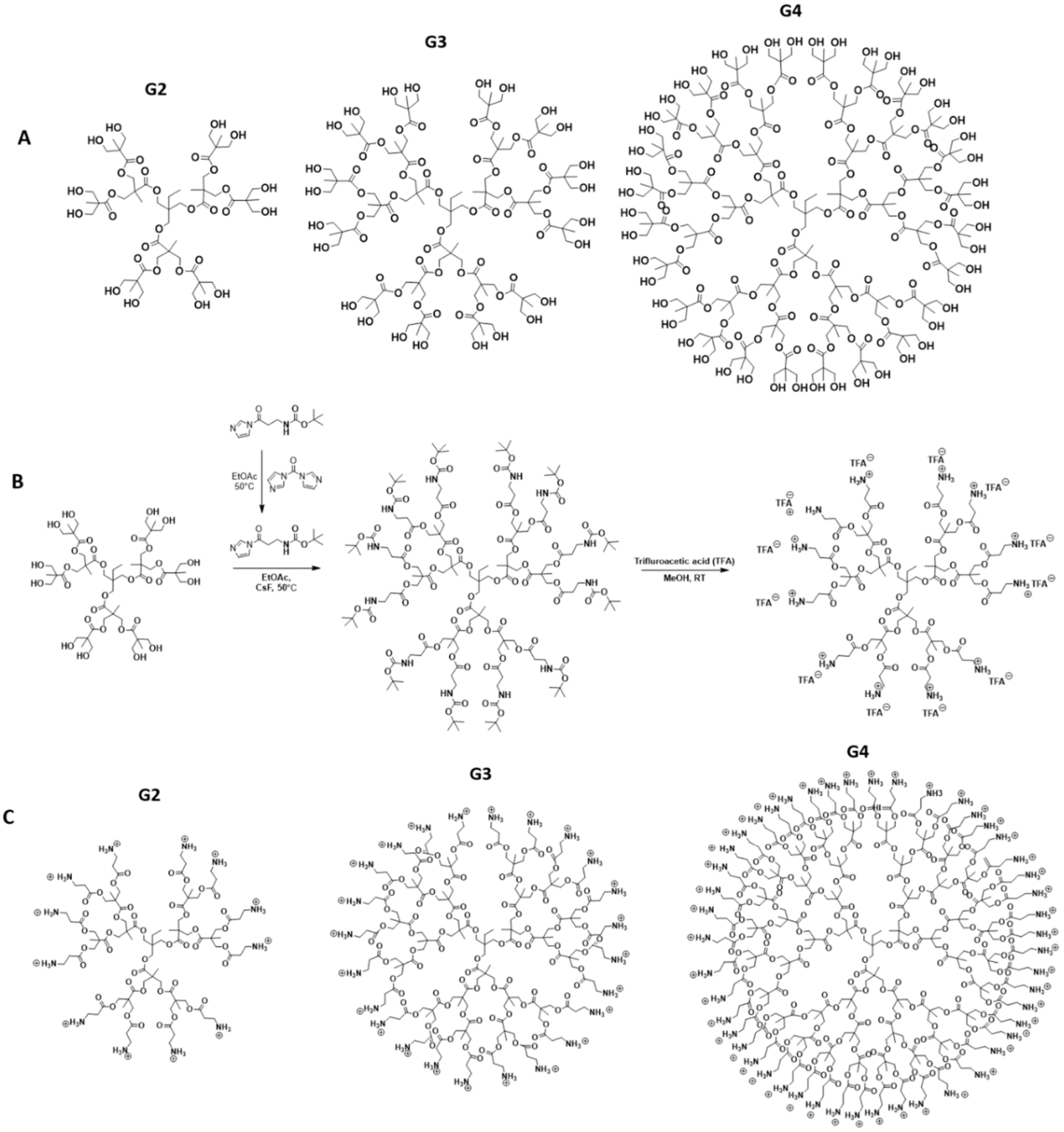

Figure S1. A) Structures of hydroxy functional dendrimers from G2 to G4. B) Synthetic procedures for the cationic dendrimer (G3). C) Structures of cationic dendrimers from G2 to G4.

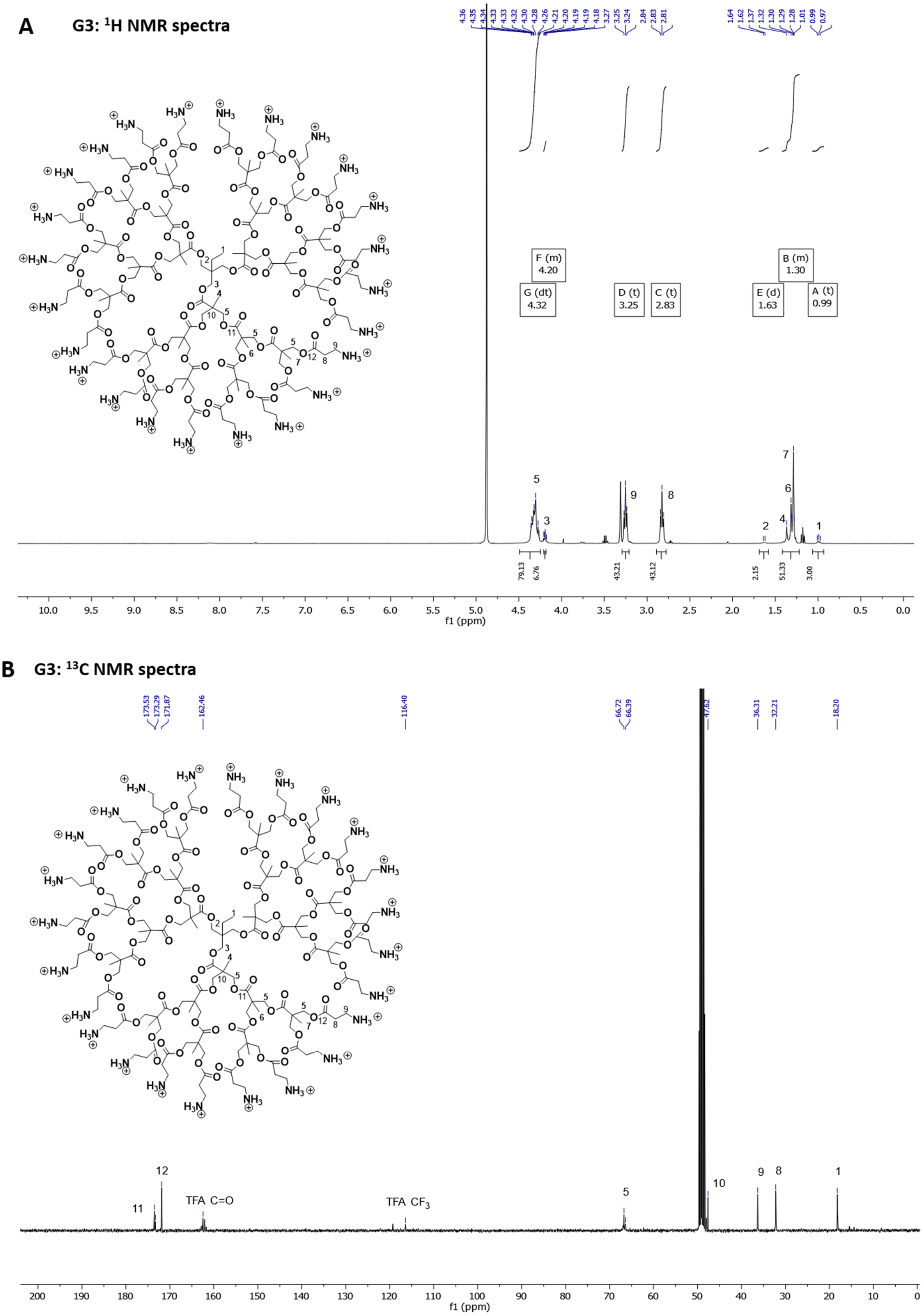

Figure S2. A) ^{1}H and B) ^{13}C NMR spectra of G3 in methanol-d_4.

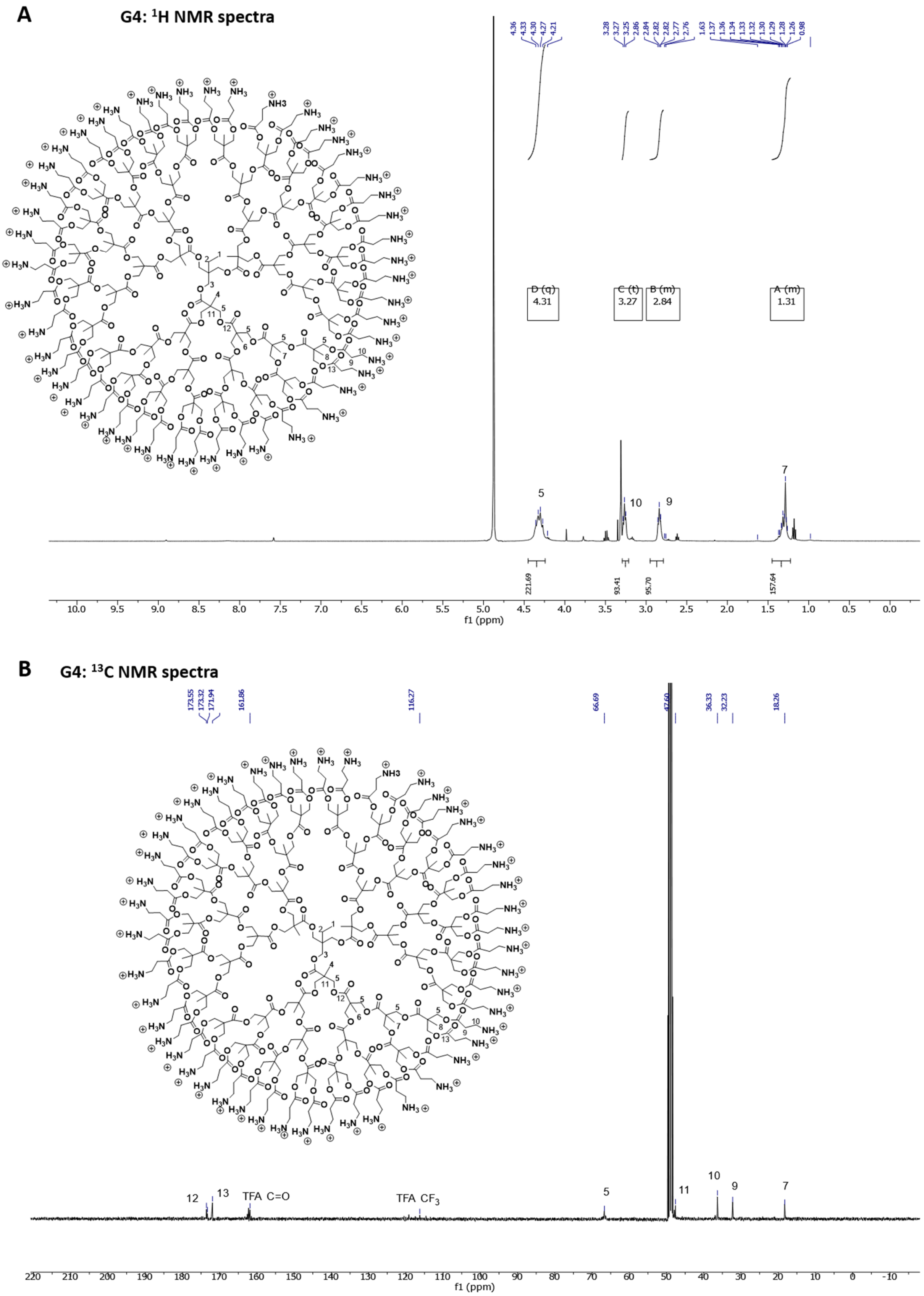

Figure S3. A) ^{1}H and B) ^{13}C NMR spectra of G4 in methanol-d_4.

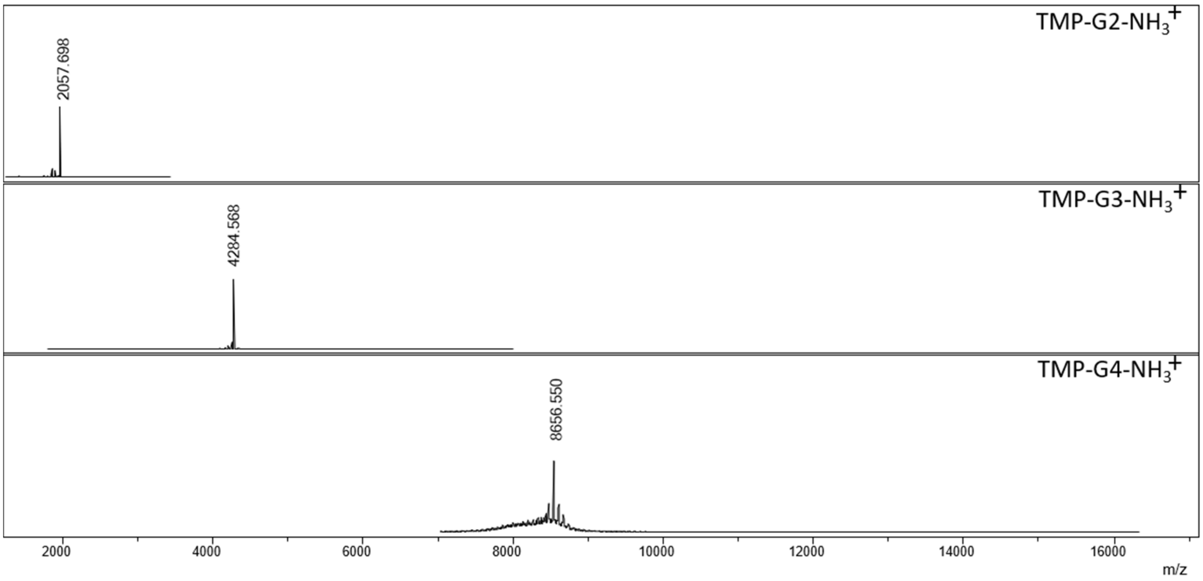

Figure S4. MALDI-TOF spectra of cationic dendrimers from G2 to G4.

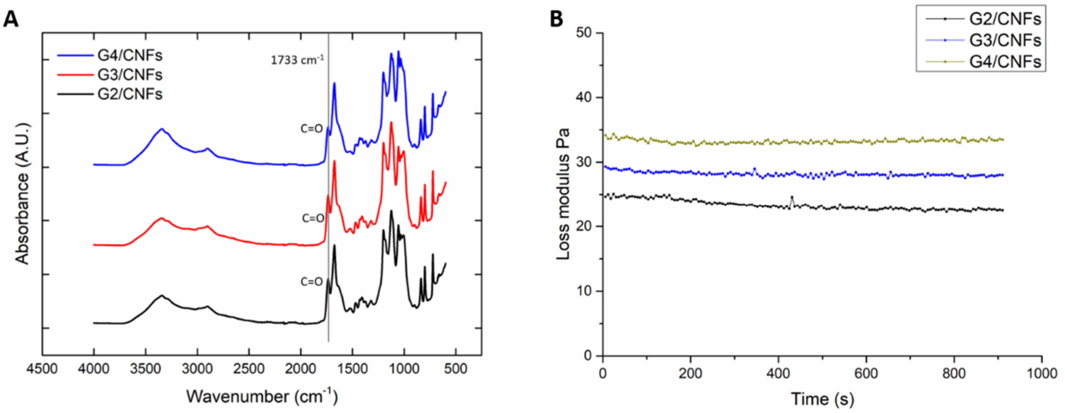

Figure S5. A) FTIR spectra of G2/CNFs, G3/CNFs and G4/CNFs showing dendrimer peak at 1733 cm^{-1}. B) The loss moduli of the thin-film hydrogels of G2/CNFs, G3/CNFs and G4/CNFs from the rheology evaluations.

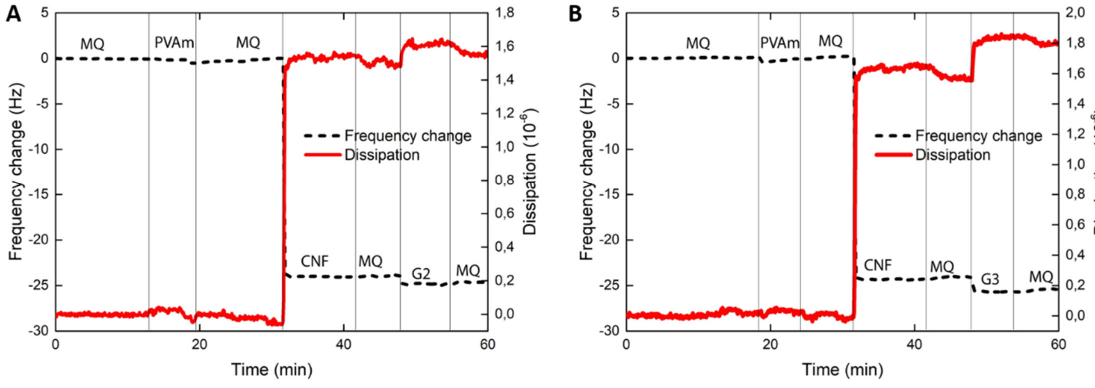

Figure S6. QCM-D of the interaction of CNFs with A) G2 and B) G3.

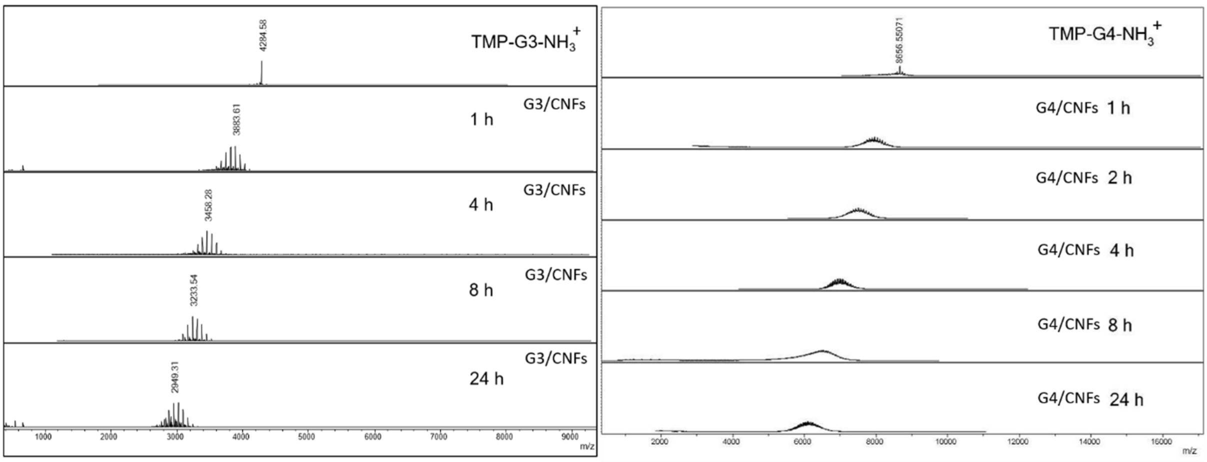

Figure S7. Leaching out study of G3/CNFs and G4/CNFs using MALDI-TOF.

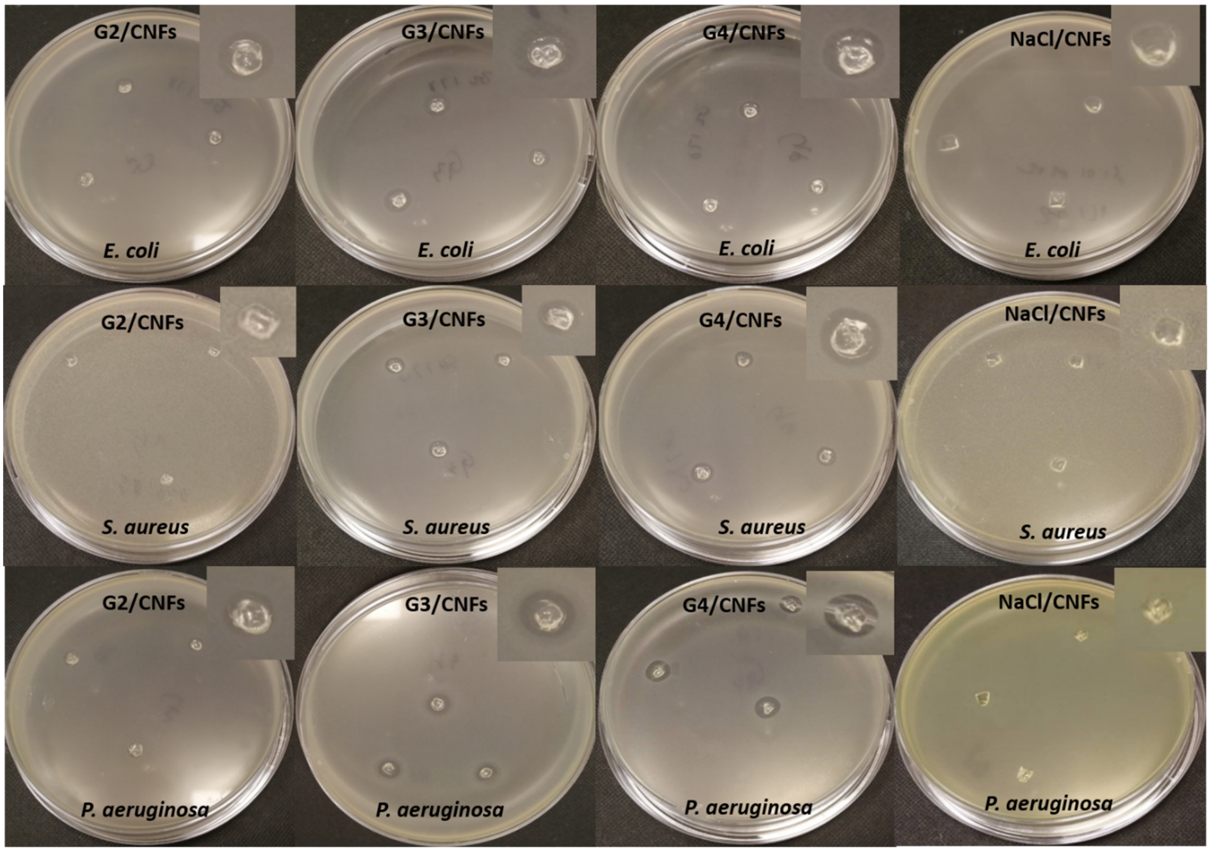

Figure S8. The disk diffusion test of the hybrid hydrogels using bacterial concentration of 10^7 CFU/mL, and NaCl/CNFs hydrogels were used as control.

Reference

1. Saito T, Hirota M, Tamura N, Kimura S, Fukuzumi H, Heux L, Isogai A. Individualization of nano-sized plant cellulose fibrils by direct surface carboxylation using TEMPO catalyst under neutral conditions. *Biomacromolecules*. **2009**, *10*, 1992–1996,doi:10.1021/bm900414t.
2. Kaldéus T, Nordenström M, Carlmark A, Wågberg L, Malmström E. Insights into the EDC-mediated PEGylation of cellulose nanofibrils and their colloidal stability. *Carbohydr. Polym*. **2018**, *181*, 871–878,doi:10.1016/j.carbpol.2017.11.065.
